# Esthetic Assessment Succeeding Anterior Atrophic Maxilla Augmentation with Cancellous Bone-Block Allograft and Late Restoration Loading

**DOI:** 10.3390/jcm10204635

**Published:** 2021-10-09

**Authors:** Sarit Naishlos, Eran Zenziper, Helena Zelikman, Joseph Nissan, Shaked Mizrahi, Gavriel Chaushu, Shlomo Matalon, Liat Chaushu

**Affiliations:** 1 Department of Pedodontology, School of Dental Medicine, Tel Aviv University, Tel-Aviv 6997801, Israel; river554@gmail.com; 2 Department of Oral Rehabilitation, The Maurice and Gabriela Goldschleger School of Dental Medicine, Tel Aviv University, Tel-Aviv 6997801, Israel; eranzen@gmail.com (E.Z.); helenapl@gmail.com (H.Z.); nissandr@gmail.com (J.N.); shakedmizrahi1993@gmail.com (S.M.); matalons@tauex.tau.ac.il (S.M.); 3 Department of Oral and Maxillofacial Surgery, School of Dental Medicine, Tel Aviv University, Tel-Aviv 6997801, Israel; 4 Department of Periodontology and Implant Dentistry, School of Dental Medicine, Tel Aviv University, Tel-Aviv 6997801, Israel; liat.natanel@gmail.com

**Keywords:** bone-block, allograft, esthetics

## Abstract

Background: Various conditions may lead to bony deficiency in the anterior maxilla. The present study evaluated esthetic (PES—pink esthetic score and WES—white esthetic score) results after augmentation of the anterior atrophic maxilla using cancellous bone-block allograft followed by implant placement and late (conventional) loading. Methods: Cohort study that included 33 patients with missing teeth in the upper anterior region characterized by extensive bone loss. Allogeneic cancellous bone-blocks were used for augmentation. Six months later, a dental implant was inserted. After a waiting time of an additional six-months, implant exposure and reconstruction were performed. The mean follow-up period was 62.93 ± 17.37 months (range 19–82 months). Results: The mean value of PES/WES was 17.8 ± 2.78. All patients had a PES/WES value above 12 (threshold value defined as clinically acceptable esthetics). The mean value of PES was 9.0 ± 1.79 and the mean value of WES was 8.8 ±1.84. Conclusions: Bone augmentation of the anterior atrophic maxilla using cancellous block-allograft and late loading supports achievement of a predictable esthetic result with long-term stability of soft and hard tissues around implant-supported reconstructions.

## 1. Introduction

The thickness of buccal bone in the anterior maxilla is considered an important factor in the long-term restoration esthetics [1]. Various conditions may lead to bony deficiency in the anterior maxilla such as congenital missing teeth, trauma, tooth extraction or periodontal disease. The use of different bone substitutes in atrophic regions has led to different results in the level of bony support of the soft and hard tissues around dental implants [2]. In extensive atrophic cases, the use of bone block is recommended [3]. The bone block may provide three-dimensional stability to support the soft tissue.

Extra-oral or intra-oral autograft may be considered the gold standard for bone block in atrophic cases requiring bony augmentation. Autografts have a number of drawbacks including postoperative morbidity (high risk of vascular or neurological damage at the harvest site, patient discomfort), limited bone availability, surgery time and bone resorption [4,5,6]. In recent years, the use of allograft is increasing. These bone blocks undergo thermal and chemical manipulations that clear them of components that can elicit potential immunological responses in the graft recipient [7]. There are different types of allogeneic blocks; cancellous without cortical layer and cortical or cortico-cancellous containing cortical layer [7]. An important advantage of allograft blocks is that harvesting does not significantly increase surgery time. There is also a decrease in postoperative morbidity. Additional benefits include initial block design capability and unlimited bone availability [8]. Another significant advantage of block-allografts is lower resorption (up to about 10%) vs. autografts (up to about 50%) between grafting and implant placement [8,9,10].

The final esthetic result is dictated by several variables [11]. Surgical variables; three-dimensional placement of the implant within the bony envelope; satisfactory reconstruction of bone in terms of height; thickness of the buccal wall. Prosthetic variables; quality of the restoration itself; support of the soft tissues surrounding the implant. The optimal location of a dental implant is in the center of the ridge. The average thickness of the buccal bone around the implant should be in the range of 2–4 mm [12]. In order for an implant to be considered successful it must meet patient satisfaction from the esthetic aspect [13].

An objective assessment of the esthetic result obtained in anterior-supported implant restoration is usually done using two PES (pink esthetic score) and WES (white esthetic score) indices. All parameters are tested in comparison to the homologous tooth [14,15]. The PES index describes an evaluation of the soft tissue around a single implant according to five parameters: (1) The presence/absence of the mesial papillae; (2) the presence/absence of the distal papillae (3) curvature contours of the buccal gums; (4) height of the buccal gums; (5) curvature of the root/color and the texture of the soft tissues in the buccal aspect of the implant site [15]. The WES index examines five parameters that relate to the evaluation of the reconstruction/visible part: (1) The general shape of the teeth; (2) outline and volume of the clinical crown; (3) color, which includes the evaluation of the hue and value indices; (4) surface texture; (5) transparency and characterization [16].

The use of late (conventional) loading allows verification of the implant osseointegration and satisfactory healing of the soft and hard tissues [17]. In recent years, due to the rising demand for shortening recovery time and treatment, especially in esthetic frontal areas of the maxilla, the immediate loading (at the end of implant placement the reconstruction is installed) method has been developed. The advantages are reduction in the number of surgical operations while restoring immediate function and esthetics. At the same time, there are risks of loading an implant that has not yet undergone osseointegration, and in addition, there is a lack of control over the final location of the soft and hard tissues, that have not yet undergone initial healing [18].

The purpose of the present study was to evaluate the esthetic results, according to WES, PES parameters, following alveolar ridge augmentation of the anterior atrophic maxilla using cancellous bone-block allograft, implant placement and late (conventional) loading. The null hypothesis was that a high esthetic score would be obtained thanks to block-grafting and late loading, which would allow better support and control of the soft and hard tissues.

## 2. Materials and Methods

The study was approved by the Ethics Committee of Tel Aviv University no. 2137-1. A retrospective photographic study that included 33 patients with a missing tooth in the upper anterior region characterized by extensive bony deficiency (≥3 mm horizontal and/or ≥3 mm vertical) according to primary cone beam computerized tomography (CBCT). Alveolar ridges were augmented using allogeneic cancellous bone-blocks. Six months later, implant placement was performed and after a six-month osseointegration waiting time, exposure and reconstruction was performed.

The patients were treated at Tel Aviv University between the years 2004–2010 by an oral rehabilitation specialist—J.N., and an oral and maxillofacial surgeon—G.C. Once a year radiological, clinical and photographic follow-up was performed. Mean follow-up was 62.93 ± 17.37 months (range 19–82 months).

Inclusion criteria:

1. Age ≥ 18 years.
2. Missing tooth in the maxillary esthetic area (between canines).
3. Bone augmentation performed 6 months prior to implant placement (using allogeneic bone block).
4. Implant loading 6 months after placement.

Exclusion criteria:

1. Patients with unbalanced systemic diseases (e.g., uncontrolled diabetes).
2. History of radiation to the head and neck area.
3. Poor oral hygiene.
4. Mucosal diseases (lichen planus).
5. Para-function (bruxism).
6. Using bisphosphonates, calcium channels blockers, epileptic drugs.
7. Patients with immunosuppression.
8. Untreated and uncontrolled periodontal disease.
9. Heavy smoking (over 10 cigarettes a day).

All patients underwent initial preparation that included oral hygiene instructions and initial preparation.

PES index was used to perform soft tissue assessments. PES is mainly affected by the local anatomy and the surgical procedure used to repair the bone defects that routinely appear at the implant site after tooth extraction.

1. The presence/absence of mesial papillae
2. The presence/absence of the distal papillae
3. Curvature contours of the buccal gums
4. Height of the buccal gums
5. Root convexity/color and soft tissue texture in the buccal aspect of the implant site [15].

Each parameter can have a score of 0, 1, or 2 [15]. The score on the mesial and distal papillae is given as a reference to the level of papillary presence. Two points will be given to a situation where there is a full papilla, 1 point will be given to a partial papilla, and a lack of papillary tissue will receive a score of 0. The curvature of the buccal gingiva, which is also defined as the emerging profile of the soft tissue reconstruction profile, is estimated as; whether it is the same (2 points), slightly different (1 point) or completely different (0 points) compared to the natural homologous tooth for the implant (as a review). The height of the buccal gingiva is estimated in comparison with the contralateral tooth as; whether there is a similar vertical height (2 points), slight mismatch (smaller or equal to 1 mm, 1 point) or significant mismatch (larger or equal to 1 mm, 0 points). The fifth and final parameter combines 3 different variables—the root/color curvature and the soft tissue texture in the buccal aspect of the implant site. In order to get a full score (2 points), the three variables must be similar compared to the homologous tooth. One point will be given in case that 2 variables out of the 3 are found, and 0 points will be given to the case where none or only 1 of the variables is found compared to the homologous tooth. The highest result that can be obtained in the index is 10 [15].

In order to evaluate the reconstruction itself, the WES index is used, which is mainly influenced by the laboratory work and the assessment of the attending physician. As a reference, a comparison of the restoration to the natural homologous tooth is made, if possible [15].

This index examines 5 parameters:

1. The shape of the original tooth;
2. Outline and volume of the clinical crown;
3. A color that includes the evaluation of hue and value indices;
4. Surface texture;
5. Transparency and characterization.

Each parameter receives a score of 0, 1, or 2, and the highest result that can be obtained in the index is 10. The highest score that can be obtained in the PES/WES composite index is 20 which shows as high an esthetic fit as possible of the restored tooth on the implant and the soft tissues surrounding it [15]. The minimum threshold value defined as clinically acceptable for the PES or WES index is 6, and in addition, the total threshold value for PES + WES that is clinically acceptable is 12 [15].

### Statistical Analysis

Due to the rare inclusion criteria (single tooth missing, extensive bone loss, block augmentation) the number of subjects was limited. Consequently, all patients corresponding to the inclusion criteria were included and no sample size calculation was performed. The statistical analysis was performed using SPSS software version 24.0 (SPSS Inc., Chicago, IL, USA) -STATA 15.1, StataCorp LLC, College Station, TX, USA). A *p*-value less than 0.05 was considered as statistically significant. Descriptive statistics were used for study participants and characteristics related to esthetic soft tissue and prosthetic rehabilitation analysis. Esthetic criteria (PES, WES) were averaged for statistical examination. Evaluation of patient demographics (age, gender, etiology, tooth involved, implant brand) as factors that may affect esthetic parameters was performed using multivariate analysis.

## 3. Results

### 3.1. Demographic Data

The study included 10 men and 23 women. Their average age was 38.2 ± 18.59 years (range 18–70). The average follow-up period for rehabilitation was 62.9 ± 17.37 months (range 19–82 months). The dental areas where the implants were performed were; tooth 11 (18/33 of the patients), tooth 12 (6/33 of the patients), tooth 21 (3/33 of the patients), tooth 22 (5/33 of the patients), and tooth 23 (1/33 of the patients). The etiology of bone deficiency was; periodontal patients (14/33), trauma patients (15/33), and congenital missing teeth patients (4/33). All implants were inserted at the bone level with 23/33 of the implants from MIS (MIS Implants Technologies, Bar Lev Industrial Center, Israel), 9/33 of the implants from ZIMMER (ZIMMER Biomet, Warsaw, Indiana, USA) and 1/33 of the implants from 3I (Implant 3I Innovations, Palm Beach Gardens, FL, USA).

### 3.2. Detailed PES and WES Values of All Patients

The detailed table of PES and WES values includes 33 patients (Table 1). The total minimum threshold value for clinically acceptable PES + WES is 12 [15]. Of the 33 patients, there were no patients in this study who presented a weighted sum of PES + WES below 12, so esthetically there are no results that are below the clinically acceptable threshold value.

### 3.3. Analysis of Esthetic Results by Index: PES

**Figure 1:**
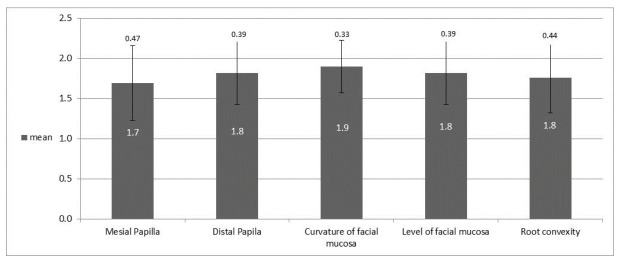
Mean PES value of each parameter indicating a good pink esthetic result.

The total average value of the PES index was 9.0 ± 1.79 (range 5–10). In addition, relatively similar and high results were found in all the parameters examined (Figure 1); mesial papillae (1.7 ± 0.47), distal papillae (1.8 ± 0.39), buccal gum contour curvature (1.9 ± 0.33), buccal gum height (1.8 ± 0.39), root/color curvature and soft tissue texture in the buccal aspect of the implant site (1.8 ± 0.44). The minimum threshold value defined as clinically acceptable for the PES or WES index is 6 [15]. In 29/33 implant sites (87.87%), PES values were obtained above the minimum threshold value, which generally indicates a good esthetic result.

### 3.4. Analysis of Esthetic Results by Index: WES

**Figure 2:**
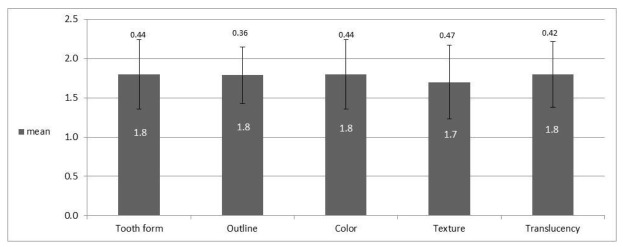
Mean WES value of each parameter indicating a good white esthetic result.

The mean value of the WES index was (8.8 ± 1.84) with a range of 5–10. Also, relatively similar results were found in the parameters examined (Figure 2); the original tooth shape (1.8 ± 0.44), outline and volume of the clinical crown (1.8 ± 0.36), color including the evaluation of hue and value indices (1.8 ± 0.44), surface texture (1.7 ± 0.47), transparency and characterization (1.8 ± 0.42). A total of 30/33 crowns tested (90.9%) received a total WES value above 6, which is the clinically acceptable threshold value.

### 3.5. Analysis of Esthetic Results by Index: PES and WES

**Figure 3:**
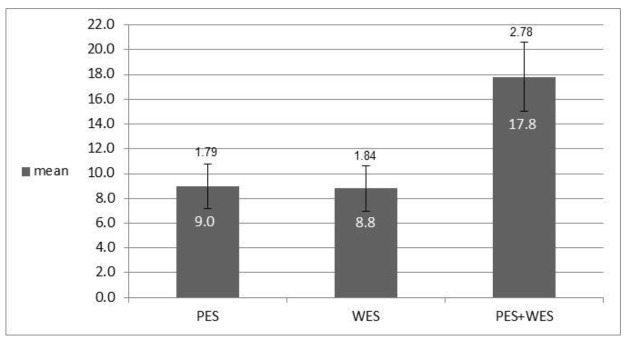
Mean total value PES and WES indicating a good total esthetic result.

**Figure 4:**
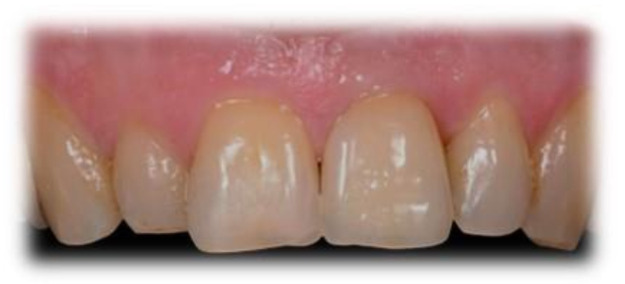
Tooth number 21, 59-month follow-up: PES = 9, WES = 9.

**Figure 5:**
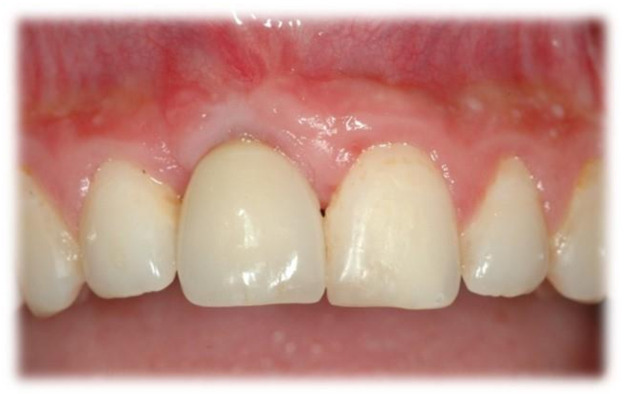
Tooth number 11, 49-month follow-up: PES = 7, WES = 7.

In the combined esthetic results analysis, according to the PES and WES indices (Figure 3, Figure 4 and Figure 5), it was found that the total PES average (9.0 ± 1.79) was higher than the average of the overall WES index (8.8 ± 1.84) and that the weighted average of both was 17.8 ± 2.78.

### 3.6. Multivariate Analysis

Multivariate analysis evaluated patient demographics (age, gender, etiology, tooth involved, implant brand) as factors that may affect esthetic parameters. There was no statistically significant effect on the esthetic parameters evaluated.

## 4. Discussion

The anterior maxilla is a challenging area, with high demand for esthetics [19,20]. The peri-implant bony support and soft tissue dimensions are considered interrelated key factors, dictating the esthetic outcome [21,22].

The position of the dental implant in relation to the neighboring teeth and in relation to the buccolingual dimension of the alveolar ridge are factors that have been proven to affect the level of bony support over the years [23]. In order to maintain bone height and prevent long-term receding gums, it is recommended to have at least 2 mm of buccal alveolar bone at the neck of the implant [12,23,24]. Insufficient thickness of the buccal bone can lead to loss of buccal bone height, followed by soft tissue recession leading to compromised biomechanical and esthetic results [25,26]. In order to overcome a challenge such as extensive bone loss, many augmentation techniques have been developed over the years that have been proven to be effective, one of which is the use of allogeneic bone blocks [10].

In 2005, the “pink esthetic score” index was introduced by Fürhauser et al. and focused on soft tissue aspects of anterior implants. In 2009, Belser et al. [15] introduced the improved esthetic index PES/WES which is a variation that incorporates the previous definitions along with the addition of the white esthetic index. The researchers defined that the clinically acceptable total threshold for PES + WES was 12 [15].

In the present study, the esthetic result was measured using the PES and WES indices 15. The overall mean score obtained in the PES/WES indices was 17.8 ± 2.78 (with a result range of 12–20). All patients included in the study received a PES/WES value above 12, which supports the fact that the final esthetic result obtained in the study was successful and satisfactory. The minimum threshold value defined as clinically acceptable for a single index (PES or WES) is 6 [15]. The overall result obtained in the present study in the PES index was 9.0 ± 1.79 (after a mean follow-up period of 62.93 ± 17.37 months), which was slightly higher than the results obtained by Yildiz et al. (2018) [27], who reported a PES index average of 8.35 at one year follow-up in the “late loading” group. The result of the present study can be explained by the fact that thanks to the amount of buccal bone created with the use of an allogeneic cancellous bone-block, not only was a stable and esthetic result of soft tissue around the implant achieved, but also the result was maintained over time (average follow-up >5 years). Another explanation lies in the fact that all the patients included in the study came to maintenance sessions at least once a year and thus a continuous and appropriate follow-up was ensured, minimizing the risk of long-term complications.

These results are consistent with the results of the study by Nissan et al. (2011) who showed that after augmentation of an allograft bone block in atrophic anterior maxilla, an average bone gain of 5 ± 0.5 mm in the horizontal dimension and 2 ± 0.5 mm in the vertical dimension was achieved. In addition, the mean buccal bone thickness observed near the neck of the implant was 2.5 ± 0.5 mm [8].

The mean value of the WES index in the present study was (8.8 ± 1.84), as shown in Figure 3. The WES index is mainly affected by the laboratory work and the review of the prosthodontist [15]. In the present study, one laboratory was used for all patients and the reconstruction was performed by one prosthodontist (permanent reconstruction of a metal-free crown).

The PES/WES indices in the present study are high compared to studies using the immediate loading method. Mangano et al. (2016) [28] presented in their study that in the group where late implant placement with immediate loading was used, the PES index was 7.4 ± 1.8, the WES index was 7.8 ± 2.1 and the overall PES/WES index was 15.2 ± 3.3, after 3 years. In addition, in a study by Yildiz et al. in 2018 [27], it was found that the result of the PES index in the “immediate loading” group was 8.2 after about a year. Other studies have examined the esthetic evaluation of reconstructions on implants in the anterior maxillary region and reported PES/WES results of 14.7 ± 1.8 [15] and 14.44 ± 2.34 [29], however, these studies were performed using a protocol of extraction and immediate implant placement with simultaneous bone grafting.

The better scores in the present study can be attributed to the use of late loading (conventional) [17,19]. The results of the present study are consistent with the null hypothesis that an adequate esthetic result would be obtained thanks to the use of cancellous bone-block allograft with delayed implant placement.

Limitations of the current study; limited number of participants, retrospective, only one treatment center, only one augmentation material. Strengths of the present study; the mean follow-up period of 62.93 ± 17.37 months (range 19–82 months), the ability to see the individual scores for each tooth. Further studies are needed to validate the effect of using allograft bone-blocks on the esthetic results adopting a late (conventional) loading protocol.

## 5. Conclusions

It can be concluded that the use of cancellous bone-block allograft and late loading, permits a predictable esthetic result and stability of the peri-implant soft and hard tissues over time.

## Figures and Tables

**Table 1:** Detailed PES and WES values of all patients.

Patient	Implant Site	PES						WES						Total
		Mesial Papilla	Distal Papilla	Curvature of Facial Mucosa	Level of Facial Mucosa	Root Convexity	Total	Tooth Form	Outline	Color	Texture	Translucency	Total	
1	11	1	1	1	1	1	**5**	2	2	2	2	2	**10**	15
2	11	1	1	1	1	1	**5**	2	2	2	2	2	**10**	15
3	12	2	2	2	2	2	**10**	2	2	2	2	2	**10**	20
4	11	2	2	2	2	2	**10**	2	2	2	2	2	**10**	20
5	11	2	2	2	2	2	**10**	2	2	2	2	2	**10**	20
6	11	2	2	2	2	2	**10**	2	2	2	2	2	**10**	20
7	11	1	1	1	1	1	**5**	1	2	1	1	2	**7**	12
8	11	1	1	1	1	1	**5**	2	2	2	2	2	**10**	15
9	11	2	2	2	2	1	**9**	2	2	2	2	2	**10**	19
10	11	2	2	2	2	2	**10**	2	2	2	2	2	**10**	20
11	11	2	2	2	2	2	**10**	2	2	2	2	2	**10**	20
12	11	2	2	2	2	2	**10**	2	2	2	2	2	**10**	20
13	11	2	2	2	2	2	**10**	1	1	1	1	1	**5**	15
14	11	2	2	2	2	2	**10**	1	2	1	1	1	**6**	16
15	11	2	2	2	2	1	**9**	1	2	1	1	1	**6**	15
16	11	2	2	2	2	2	**10**	2	2	2	2	2	**10**	20
17	11	2	2	2	2	2	**10**	2	2	2	1	2	**9**	19
18	11	2	2	2	2	2	**10**	2	2	2	2	2	**10**	20
19	21	1	1	2	1	1	**6**	1	1	2	1	1	**6**	12
20	11	2	2	2	2	2	**10**	2	2	2	2	2	**10**	20
21	21	2	2	2	2	2	**10**	2	2	2	2	2	**10**	20
22	22	1	2	2	2	2	**9**	1	1	1	1	1	**5**	14
23	12	1	2	2	2	2	**9**	2	2	1	2	2	**9**	18
24	22	2	2	2	2	2	**10**	2	2	2	2	2	**10**	20
25	12	2	2	2	2	2	**10**	1	1	1	1	1	**5**	15
26	21	2	2	2	2	2	**10**	2	2	2	1	2	**9**	19
27	22	2	2	2	2	2	**10**	2	2	2	1	2	**9**	19
28	12	2	2	2	2	2	**10**	2	2	2	2	2	**10**	20
29	23	1	1	2	1	1	**6**	1	1	1	2	1	**6**	12
30	22	1	2	2	2	2	**9**	2	2	2	2	2	**10**	19
31	12	1	2	2	2	2	**9**	2	2	2	2	2	**10**	19
32	22	2	2	2	2	2	**10**	2	2	2	2	2	**10**	20
33	12	2	2	2	2	2	**10**	2	2	2	2	2	**10**	20
**Mean**		**1.7**	**1.8**	**1.9**	**1.8**	**1.8**	**9**	**1.8**	**1.8**	**1.8**	**1.7**	**1.8**	**8.8**	17.8

## Data Availability

Data supporting reported results can be found in the table.
